# Greater impulsivity is associated with a reduced propensity to cash out of bets

**DOI:** 10.1016/j.abrep.2025.100645

**Published:** 2025-11-26

**Authors:** Ong George Ngieng, Lucy Albertella, Ty Hayes, Antonio Verdejo-Garcia, Lukasz Walasek, Elliot A. Ludvig, Daniel Bennett

**Affiliations:** a School of Psychological Sciences, Monash University, Australia; b Warwick Business School, University of Warwick, UK; c Department of Psychology, University of Warwick, UK; d Melbourne School of Psychological Sciences, The University of Melbourne, Australia

**Keywords:** Cognitive inflexibility, Impulsivity, Instant cash-out, Sensation seeking, Sports betting, Urgency

## Abstract

A common feature of contemporary sports-betting apps is ‘instant cash-out’, which allows users to settle a bet early in exchange for a discounted immediate payout. Despite high prevalence and links with gambling-related harm, relatively little is known about how personality traits associated with gambling, such as impulsivity, predict instant cash-out usage. To address this question, we recruited 145 general-population adult participants (69 men, 66 women, 10 non-binary or undisclosed; *M_age_ =* 36.3, *SD* = 10.7; participants resided in Australia, Canada, Ireland, New Zealand, the UK, or the USA) to complete five self-report questionnaires related to impulsivity, as well as the Problem Gambling Severity Index (PGSI), and a validated cognitive task measuring individual differences in cash-out frequency. We then assessed how cash-out frequency in the behavioral task was associated with both self-reported impulsivity and PGSI. We found that cash-out frequency was negatively correlated both with PGSI scores and with a number of impulsivity-related traits including Dysfunctional Impulsivity, Lack of Premeditation, Positive Urgency, Sensation Seeking, and Fun Seeking. An exploratory factor analysis revealed that higher scores on a latent ‘Dysfunctional Impulsivity’ factor were negatively associated with cash-out frequency overall, whereas higher scores on an ‘Inhibition and Inflexibility’ factor predicted higher cash-out frequency specifically for bets with a low win probability. Taken together, results suggest that instant cash-out may primarily appeal to less impulsive people and those with lower PGSI scores. This raises the possibility that instant cash-out may specifically facilitate increased gambling behaviors among people with less prior experience of gambling.

## Highlights

• People who are less impulsive are more likely to cash out of a risky bet.
• People with more gambling-related harm are less likely to cash out of a risky bet.
• Greater cognitive inflexibility predicts higher cash-out rates for losing bets only.

## 1 Introduction

In the past two decades, gambling operators have developed new interactive gambling products that capitalize on the novel capabilities afforded by online platforms (Gainsbury, 2012, Hing et al., 2022). In the domain of sports betting, online gambling has changed the structural characteristics of gambling products in a way that reinforces continuous user engagement and immersion (McCormack and Griffiths, 2013, Newall et al., 2021), thereby facilitating impulsive and unplanned gambling behaviors (Lopez-Gonzalez et al., 2019). Accordingly, online sports betting products may specifically confer increased risks upon more impulsive users of gambling products (Russell et al., 2019). Here, we investigated this proposition in the context of *instant cash-out*, a widespread in-play gambling product that is offered on most major sports-betting apps and websites (Lopez-Gonzalez and Griffiths, 2017, Sinclair et al., 2024).

Instant cash-out is an interactive betting feature that gives users the ability to cancel their bet during a sporting event or a race in exchange for an immediate payout (Bennett et al., 2024, Grant et al., 2021, Sinclair et al., 2024). The amount of this immediate payout (the ‘cash-out offer’) is proportional to the momentary expected value of the bet, meaning that it changes over time within an event as the bet’s probability of paying out changes. As such, users can strategically cash out either to avoid losses in bets with deteriorating payout odds or to lock in ‘paper gains’ in bets with improving odds. Importantly, however, cashing out is financially disadvantageous because cash-out offers typically include a built-in profit margin for the bookmaker (Newall & Cortis, 2019), meaning that users who cash out frequently will tend to see lower average returns from their bets. Nevertheless, a recent study found that a majority of online sports-betting users report having used instant cash-out (Sinclair et al., 2024) and, concerningly, that users of instant cash-out reported higher levels of gambling-related harm and psychological distress than non-users. Parke & Parke (2019) suggest that instant cash-out may confer risk for gambling harm by prolonging sessions of online sports-betting, because funds that are cashed out early from one bet are immediately available to be re-staked on another. Separately, a recent experimental study found that the mere availability of instant cash-out within a behavioral gambling task significantly increased participants’ average wager amounts (Bennett et al., 2024). Bennett et al. also found that there were marked differences in cash-out frequency between participants, with the tendency for cash-out availability to inflate wagers strongest among participants who cashed out most frequently.

The associations between cash-out usage and potentially hazardous gambling behavior highlight the importance of understanding the source of inter-individual differences in rates of usage of instant cash-out. Here we sought to answer this question by examining the role played by impulsivity in decisions to cash out of a risky bet. Impulsivity is a trait broadly understood as a tendency to act on urges without forethought or consideration of potential negative consequences (DeYoung & Reuter, 2017). Higher impulsivity is associated with increased risk for gambling-related harm (Ioannidis et al., 2019, Smith et al., 2007) as well as for behavioral addictions more broadly (Verdejo-García et al., 2008), as part of a broader set of vulnerability factors including alexithymia, dissociation, and emotion dysregulation (Buen and Flack, 2021, Topino et al., 2024). Personality theorists have proposed that impulsivity is a broad ‘umbrella’ concept (Depue and Collins, 1999, Whiteside and Lynam, 2001), though there is ongoing disagreement concerning its underlying theoretical structure (e.g., Smith et al., 2007, Strickland and Johnson, 2021). Behaviorally, impulsivity is associated with a tendency to take rapid actions in pursuit of large rewards, disregarding risks or potential losses (Green & Myerson, 2010), an increased preference for immediate over delayed rewards (Moreira & Barbosa, 2019; but see van Baal et al., 2025) and a reduced capacity to inhibit prepotent behavioral responses (Logan et al., 1997). Of particular relevance to instant-cash out, greater self-reported impulsivity predicts greater engagement with in-play sports betting (Hing et al., 2018), potentially due to the facilitation of rapid and impulse-driven behavior by the structural characteristics of online sports betting (Russell et al., 2019).

How might impulsivity be related to the decision to cash out of a bet? If impulsivity is conceptualized as a disregard for the potential negative consequences of one’s actions (e.g., Lauriola et al., 2014), then more impulsive people should be less likely to seek the relative safety of instant cash-out. Conversely, if impulsivity is conceptualized as a preference for immediate over delayed rewards or reduced perseverance (e.g., Mallorquí-Bagué et al., 2019), more impulsive people should be *more* likely to accept an immediate reward available by cashing out. Here our aim was to resolve these questions by determining how individual differences in impulsivity and related traits were associated with cash-out frequency within a behavioral gambling task (Bennett et al., 2024) in a general-population sample. We recruited a general-population sample because our research question concerned impulsivity as conceptualised as a broad individual-difference trait; this approach complements previous research on the more targeted question of how impulsivity is associated with cash-out usage specifically among regular users of online gambling products (Sinclair et al., 2024). We did not have *a priori* directional hypotheses; instead, we sought to identify correlations between personality traits and cash-out frequency that were consistent across a battery of self-report measures including standard measures of impulsivity, measures of related constructs such as behavioral activation and cognitive flexibility, and a standard measure of gambling-related harm.

## 2 Method

### 2.1 Participants

We recruited 145 participants (69 men, 66 women, 10 who did not endorse a binary gender) to complete the study online via Prolific. Participants were aged between 18 and 65 (*M =* 36.3, *SD* = 10.7), resided in Australia, Canada, Ireland, New Zealand, the United Kingdom, and the United States of America, were fluent speakers of English, and did not have any uncorrected vision impairment. This sample size was determined *a priori* to give in excess of 80 % power to detect a typical correlational effect size in personality and individual-differences research (i.e., *r* = 0.24; Fraley & Marks, 2007).

Participation took approximately 30 min per participant. Each participant received a base payment of AUD $8 plus task winnings up to a maximum of AUD $2 (*M* = $1.02, *SD* = 0.08). 22 participants were excluded after attention checks embedded in the behavioral task (13 participants), the questionnaires (six participants), or both (three participants), leaving a final sample of 123 participants. This experiment received ethical approval by the Monash University Human Research Ethics Committee and all participants provided informed consent.

### 2.2 Materials and Procedure

Participants who met eligibility criteria (based on internal screening questions administered separately by Prolific) were shown advertisement text describing the study on Prolific. Participants who indicated their interest on the basis of this text were then directed to a separate webpage containing full study information, a consent form, and study materials. Participants who consented to take part in the study first completed a demographic form and a battery of self-report questionnaires, followed by the card-betting task. All study materials were presented to participants within a web browser using custom code written in JavaScript using the jsPsych package (version 7.3.1) (De Leeuw, 2015).

#### 2.2.1 Self-report instruments

Because impulsivity is a multi-faceted construct with no single consensus measurement instrument, we measured individual differences using a battery of five separate self-report instruments (89 items total). Specifically, participants completed the SUPPS-P scale (Cyders et al., 2014) and the Dickman Impulsivity Inventory (Dickman, 1990) as relatively pure measures of impulsivity, the BIS/BAS scales (Carver & White, 1994) to measure the related construct of reward sensitivity, and the EDFLIX General Flexibility subscale (Dahlgren et al., 2019) to measure the related construct of cognitive flexibility. Participants also completed Problem Gambling Severity Index (PGSI) as a measure of hazardous gambling behavior. The presentation order of questionnaires was randomized and each questionnaire included one infrequency-item attention check (e.g., “I have never used a computer”; see Zorowitz et al., 2023). More information on the composition and psychometrics of each measure is provided in the Supplementary Material.

#### 2.2.2 Behavioral task

**Fig. 1:**
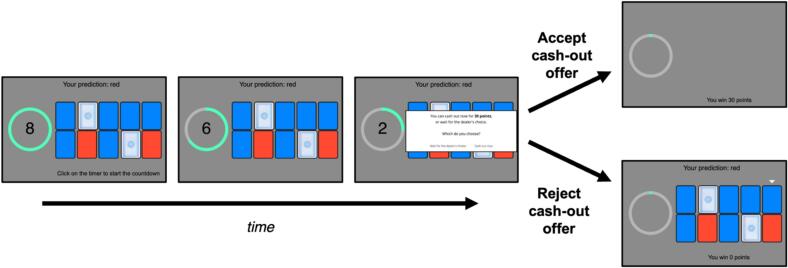
Task schematic for the card-betting task. Participants first predicted whether a blue or red card would be chosen from an as-yet-unseen array (not shown here). They were then shown the card array and a countdown timer that started when clicked by the computer mouse. Participants were given one opportunity to ‘cash out’ of their bet during the delay prior to the selection of a card. (For interpretation of the references to colour in this figure legend, the reader is referred to the web version of this article.)

The behavioral task was the ‘card-betting task’ (Bennett, 2022, Bennett et al., 2024). In each trial of this task, participants first predicted whether a red or blue card would be randomly selected from an as-yet-unseen array of ten cards after an eight-second delay (see Fig. 1). Participants won 50 points if their prediction was correct and lost 50 points if their prediction was incorrect. Participants were then shown the card array, which remained visible (and unchanged) throughout the subsequent delay period. During the delay period participants were also given one opportunity to take a ‘cash-out offer’, which was a number of points proportional to the current expected value of their prediction given the observed array. If the cash-out offer was accepted, the participant received the payout and cards were removed from the screen for the remainder of the delay. Participants were endowed with 3000 points at the start of the task, and their final bonus payment was proportional to their final points total (at a rate of AUD $1 per 3000 points). The primary dependent variable in the present study was the frequency with which different participants accepted the cash-out offer.

Participants completed 80 trials across four blocks of 20 trials each. From trial to trial we experimentally manipulated Pr(win)—the probability that a participant’s prediction would be correct—in a randomized order. Pr(win) could be either 70 % (more cards of the predicted color than the unpredicted color, and therefore a higher cash-out offer), 50 % (equal numbers of red and blue cards, moderate cash-out offer), or 30 % (fewer cards of the predicted color, lowest cash-out offer). We also manipulated the ambiguity of the initial probabilities (by varying the number of cards in the array that were face down), the generosity of the cash-out offer (which could be either 90 % or 100 % of the true expected value of the participant’s bet), and the timing of the cash-out offer during the delay period. See Bennett et al. (2024) for a more detailed description of these latter three manipulations, which were retained for consistency with previous versions of the task. Finally, the task also included eight attention-check trials in which participants were offered either an extremely high cash-out offer (100 points) or an extremely low cash-out offer (0 points). Making an incorrect response on these trials (i.e., rejecting a high cash-out offer or accepting a low cash-out offer) was taken as evidence of inattentive responding.

### 2.3 Data analysis

Analysis code and all data have been made publicly available on the Open Science Framework: https://osf.io/m9tg6. All analyses were conducted in R.

#### 2.3.1 Correlation analyses

Correlations between cash-out frequency and questionnaire subscales were estimated using non-parametric Spearman correlations because floor and ceiling effects (i.e., participants who always or never cashed out) gave cash-out frequency a non-normal distribution. To control for multiple comparisons, correlation analyses were corrected using a false discovery rate (FDR) correction (*⍺* = 0.05).

#### 2.3.2 Exploratory factor analysis

Exploratory factor analysis of item-level questionnaire responses was conducted using the *psych* package. All items were first linearly re-scaled between 0 and 1 to equate items from questionnaires with different numbers of Likert-scale response options. We screened for non-normality (absolute skew > 2 or absolute kurtosis > 7; Watkins, 2020) and estimated adequacy of the data for factor analysis using the Kaiser-Meyer-Olkin (KMO) test. 16 items with a KMO score less than 0.6 were removed prior to factor analysis. The number of factors to extract was estimated using parallel analysis, and factor analysis was conducted using a maximum-likelihood factoring method. We employed an oblique (promax) rotation method because we anticipated correlation between the latent factors.

## 3 Results

### 3.1 Cash-out frequency as a function of hazardous gambling severity

There were substantial inter-individual differences in the frequency with which participants cashed out of their bets during the task (median = 0.39, IQR = [0.24, 0.58]) as well as substantial variability in hazardous gambling behavior (as measured using the PGSI scale; mean = 1.33, SD = 2.96). When PGSI scores were categorised according to standard scoring rules, 83 participants were determined to be at “no risk” of problem gambling (PGSI = 0; 67.5 % of sample), 18 participants fell in each of the “low risk” and “moderate risk” categories (PGSI 1–4 and PGSI 5–7 respectively; 14.6 % of sample in each category) and only four participants met the criteria for high risk of problem gambling (i.e., PGSI 8+; 3.3 % of sample).

**Fig. 2:**
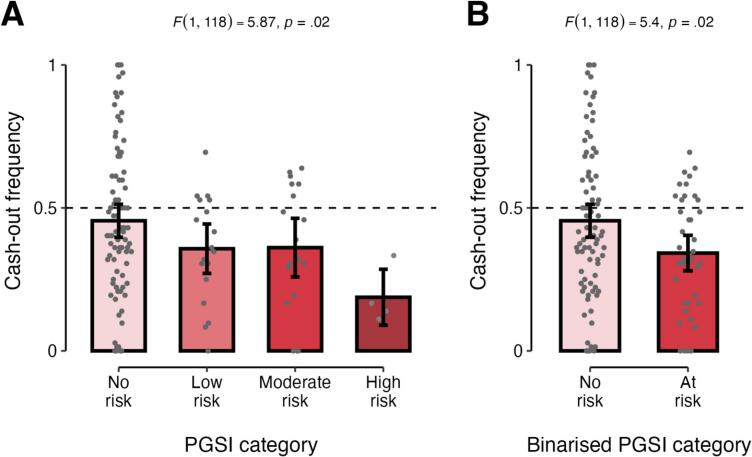
Cash-out behavior as a function of PGSI score. (A) Four-way categorisation of PGSI score according to standard cutoffs; (B) Binary categorisation of PGSI scores, with all levels of non-zero risk collapsed into a single “at risk” category. Bars represent group means and their 95% confidence intervals; background grey dots represent means of individual participants.

In analysing individual differences in behaviour, we first investigated whether individual differences in cash-out frequency were associated with individual differences in PGSI scores. A linear regression controlling for age and gender found that there was indeed an association between participants’ PGSI scores (categorized as no risk, low risk, moderate risk, or high risk according to standardized cut-offs) and their cash-out frequency (*F*(1,118) = 5.87, *p* = 0.02). Specifically, we found that participants with more severe hazardous gambling (higher PGSI scores) tended to cash out less frequently than participants not at risk (*β_PGSI_* = -0.06, *p* = 0.02; Fig. 2A) when PGSI category was coded numerically. A similar result was obtained when PGSI scores were dichotomized (*F*(1,118) = 5.40, *β_PGSI_* = -0.11, *p* = 0.02; Fig. 2B). When PGSI was treated as a nominal variable, the only significant pairwise difference in cash-out frequency between groups was between the no-risk group and the high-risk group (*β* = -0.27, *p* = 0.04).

### 3.2 Associations between self-reported impulsivity and cash-out frequency

We next investigated how cash-out frequency was related to individual differences in self-reported impulsivity and related traits. As detailed in the Supplementary Material (Table S2), all self-report scales displayed acceptable internal consistency (defined as Cronbach’s *α* > 0.7), except the Lack of Perseverance subscale of the SUPPS-P (Cronbach’s *α* = 0.69) and the BAS Fun Seeking subscale of the BIS/BAS (Cronbach’s *α* = 0.64).

**Fig. 3:**
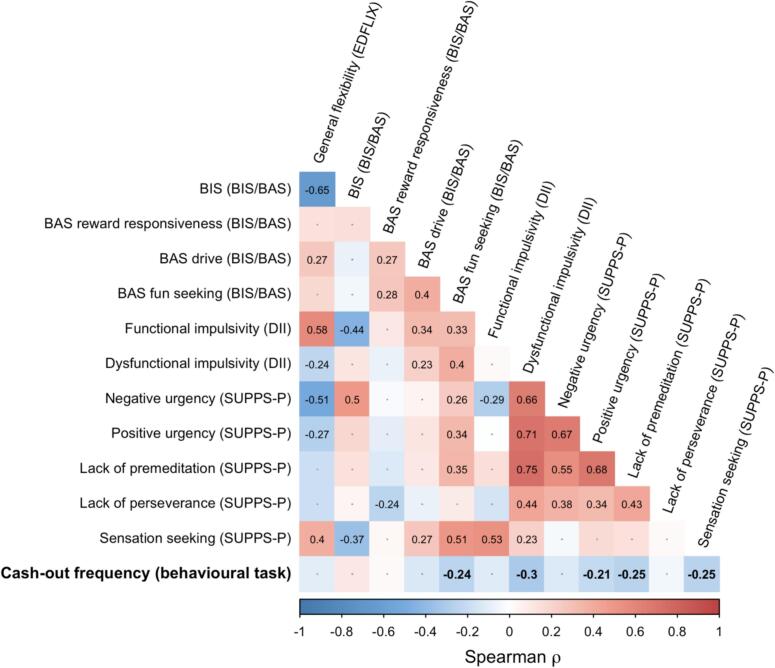
Spearman correlation matrix for associations between impulsivity subscales and overall cash-out frequency (bottom row, bold typeface). Tile color represents strength and direction of association; tiles with numerical text represent correlations that were statistically significant after FDR correction for multiple comparisons. Cash-out frequency was negatively associated with subscales related to fun seeking, dysfunctional impulsivity, positive urgency, lack of premeditation, and sensation seeking. A full correlation matrix including non-significant coefficients is presented as Supplementary Fig. S3.

Fig. 3 presents a Spearman correlation matrix for all self-report subscales and overall cash-out frequency. Overall, higher levels of impulsivity tended to be associated with a lower propensity to cash out within the behavioral task. Specifically, cash-out frequency was significantly negatively associated with the Fun Seeking scale of the BIS/BAS (ρ(121) = -0.24, *p* = 0.02), the Dysfunctional Impulsivity scale of the DII (ρ(121) = -0.30, *p* = 0.002), the Positive Urgency subscale of the SUPPS-P (ρ(121) = -0.21, *p* = 0.04), the Lack of Premeditation subscale of the SUPPS-P (ρ(121) = -0.25, *p* = 0.01), and the Sensation Seeking subscale of the SUPPS-P (ρ(121) = -0.25, *p* = 0.01) (see Supplementary Fig. S5 for scatterplots). By contrast, there were no associations between cash-out frequency and self-report scales that quantified adaptive flexibility of behavior (which might be considered a non-problematic equivalent of impulsivity) such as the General Flexibility subscale of the EDFLIX (ρ(121) = -0.11, *p* = 0.29) or the Functional Impulsivity subscale of the DII (ρ(121) = -0.12, *p* = 0.25).

The correlation between several impulsivity subscales and cash-out frequency was significantly moderated by a bet’s probability of winning. For instance, the BIS subscale of the BIS/BAS was positively correlated with cash-out frequency in the 30% Pr(win) condition only, whereas the Lack of Perseverance subscale of the SUPPS-P was negatively correlated with cash-out frequency in the 70% Pr(win) condition only (see Fig. S5 in Supplementary Material). This pattern suggests that different impulsivity-related constructs may have been differentially related to cash-out frequency in winning versus losing bets.

**Fig. 4:**
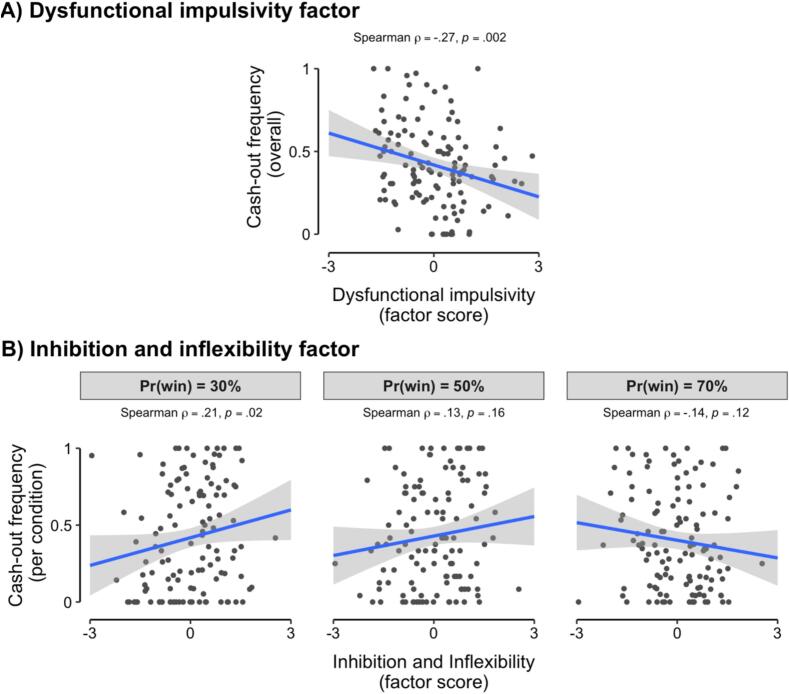
Scatterplot of participants’ cash-out frequency as a function of (A) individual differences in the ‘dysfunctional impulsivity’ factor score (x-axis; y-axis presents overall cash-out frequency averaged across all Pr(win) conditions), and (B) individual differences in the ‘inhibition and inflexibility’ factor score (x-axis; y-axis presents cash-out frequency) presented separately for each Pr(win) condition. Overlaid lines depict linear associations of best fit and their 95% confidence intervals.

Finally, responses on the different impulsivity subscales also tended to be strongly correlated with one another (upper rows of Fig. 4) and with participants’ PGSI scores (see Supplementary Table S6). These patterns of correlation indicate that participants’ responses across the different survey subscales were likely to be manifestations of a lower-dimensional latent structure of individual differences.

### 3.3 Exploratory factor analysis

The potential latent structure of participants’ responses to self-report questionnaires was assessed using an exploratory factor analysis (EFA) of item-level responses (full results provided in Supplementary Material, Section S3). This EFA produced a four-factor solution, comprising (1) a *Dysfunctional Impulsivity* factor (propensity towards impulsive actions that lead to negative consequences), (2) an *Inhibition and Inflexibility* factor (tendency towards behavioral inhibition and cognitive inflexibility), (3) a *Reward Responsiveness* factor (propensity to engage in reward- and sensation-seeking behaviors), and (4) an *Adaptability* factor (capacity to change plans when circumstances change). To investigate how behavioral cash-out frequency was associated with individual differences in the latent space defined by the extracted factors, we entered participants’ factor scores into a mixed-effects logistic regression of cash-out behavior (see Supplementary Material for full results). This analysis revealed a significant main effect of the Dysfunctional Impulsivity factor on cash-out frequency (*β* = -0.52, *p* = 0.001; Fig. 4A), with higher scores on this factor associated with lower usage of the cash-out option. There was also a significant interaction between Pr(win) and the Inhibition and Inflexibility factor (*β* = -0.45, *p* = 0.006; Fig. 4B); post-hoc analyses revealed that this interaction was driven by a significant positive effect of Inhibition and Inflexibility when Pr(win) was 30% (Spearman ρ(121) = 0.21, *p* = 0.02), but not when Pr(win) was 50% (ρ(121) = 0.13, *p* = 0.16) or 70% (ρ(121) = -0.14, *p* = 0.12).

## 4 Discussion

This study examined how individual differences in self-reported impulsivity were associated with cash-out frequency within a behavioral gambling task. There was consistent evidence for a negative correlation, meaning that participants who self-reported being less impulsive tended to accept cash-out offers more frequently within the task. This result was observed consistently across subscales from different self-report instruments (the Fun Seeking subscale of the BIS/BAS, the Dysfunctional Impulsivity subscale of the DII, and the Positive Urgency, Lack of Premeditation, and Sensation Seeking subscales of the SUPPS-P). An exploratory factor analysis further revealed a negative overall association between cash-out frequency and a latent *Dysfunctional Impulsivity* factor largely composed of items from these subscales. Taken together, these results suggest that negative associations between self-reported impulsivity and cash-out frequency are general in nature and not related to any one specific measurement instrument.

Our second primary finding was that cash-out frequency was also negatively associated with PGSI scores, meaning that individuals at lower risk of gambling-related harm tended to cash out more frequently than individuals at higher risk of harm. This finding contrasts with results reported both by Sinclair et al. (2024), who found that participants who used instant cash-out had significantly *higher* mean PGSI scores, and by Lopez-Gonzalez & Griffiths (2019), who found that participants with higher PGSI scores were more likely to report past usage of instant cash-out feature. However, a crucial distinction between our method and that of previous studies is that whereas Sinclair et al. and Lopez-Gonzalez & Griffiths specifically recruited participants who engaged in sports betting, the present study recruited participants from the general population. This distinction is important because users of online sports-betting platforms are themselves likely to be more impulsive than the general population (Killick and Griffiths, 2021, Russell et al., 2019), meaning that conditioning on online sports betting usage may give a misleading impression of the appeal of these products in the population at large (i.e., Simpson’s paradox; Hernán et al., 2011). Our results suggest that preferences for cashing out of a risky bet are negatively associated with impulsivity in the general population, even as rates of instant cash-out usage are positively associated with gambling-related harm specifically among people who frequently bet on sports (Sinclair et al., 2024).

We speculate that, in their everyday lives, less impulsive people in the general population may be less likely to use gambling products, even though they preferred cashing out more strongly when presented with the option within our behavioral task. This contrast raises the possibility that instant cash-out may serve as a ‘gateway product’ for sports-betting usage and gambling-related harm among people who would otherwise have been at lower risk of gambling-related harm. That is, the prospect of using instant cash-out to reduce the variance of gambling outcomes may specifically appeal to less-impulsive people who would not otherwise have been drawn to gambling. In line with this proposition, previous work using the card-betting task has shown that the effect of cash-out availability on betting behaviour is greatest among low-gambling-risk participants (Bennett et al., 2024). However, further research using a longitudinal design is required to determine how (and for whom) cash-out usage is related to risks for gambling-related harm over time. Such a study could, for instance, determine whether increases in people’s cash-out usage rates are a prospective risk factor for subsequent increases in their gambling-related harm). Strong positive results might even support screening for cash-out usage as a prospective risk factor for gambling-related harm by healthcare and social service professionals.

More broadly, the exploratory factor analysis of self-report instruments also revealed a second latent factor that was specifically associated with cash-out frequency in bets with a low win probability. This latent ‘Inhibition and Inflexibility’ factor measured a tendency to experience distress in response to sudden changes or events that do not go to plan; as such, this result suggests that the tendency to cash out of losing bets might be driven by participants’ negative emotional reactions to a decreasing probability of winning the bet (cf. Cholewick & Bennett, 2025), leading to loss aversion that motivates them to accept the cash-out offer (Sokol-Hessner & Rutledge, 2019). This result indicates that any overall theoretical explanation of the decision to cash out of risky bets cannot treat cash-out propensity as a unitary trait akin to risk aversion, but must instead consider how cash-out decisions are motivated by emotional and cognitive responses to different types of events as a bet unfolds.

Our findings also reveal that (un)willingness to cash out of a risky bet is a novel behavioral phenotype associated with impulsivity. This observation is theoretically noteworthy because the decision to accept a cash-out offer during a bet involves a tension between two established components of impulsive choice. On the one hand, impulsivity understood as motor disinhibition (Logan et al., 1997) or delay discounting (a preference for smaller immediate rewards over larger later rewards; Moreira & Barbosa, 2019) predicts a positive association between impulsivity and cash-out frequency, because a cash-out offer is always smaller than the potential winnings of the bet and is available immediately. On the other hand, impulsivity understood as sensation seeking that disregards potential negative consequences (e.g., Green & Myerson, 2010) predicts a negative association between impulsivity and cash-out frequency. Our results therefore support the latter model of the behavioral correlates of impulsivity.

Our results should be viewed in the light of several limitations in the study design. These limitations include the use of a laboratory gambling task to measure cash-out propensity, rather than measuring cash-out rates within actual gambling behaviour. Although this approach enabled measurement of cash-out propensity in a general-population sample, gambling behaviour in lab-based tasks often does not generalise well to real-world gambling behaviour (e.g., Anderson & Brown, 1984). Further research assessing cash-out behaviour in a naturalistic gambling setting is therefore required to test the generality of the observed effects. Moreover, the task design was less naturalistic than real-world sports-betting products: as in previous research using the card-betting task, cash-out offers were presented only once during the pre-outcome delay period, which contrasts with the near-continuous availability of cash-out offers in real-world sports-betting products. Similarly, win odds were static during the delay period in the card-betting task; this contrasts with real-world betting products, where odds fluctuate dynamically during the delay. Future research could increase the ecological validity of the task both by incorporating a fluctuating win probability and by allowing participants to cash out at any time. Finally, as discussed above, we measured cash-out propensity among a non-representative general-population sample, not among regular users of sports-betting products. This recruitment strategy enabled more nuance in our consideration of the associations between impulsivity and preferences for cashing out of a bet, but it also meant that it is an open question how our results generalise to regular users of sports-betting products. Likewise, the use of a non-representative sample means that the seemingly high proportion of participants meeting high-risk criteria for ‘problem gambling’ in our sample (3.3% according to a PGSI cut-off of 8) may not be representative of the population more broadly. This figure could be inflated by factors such as sample demographics, high rates of online technology use among participants on Prolific, as well as cohort effects such as increased post-COVID participation within online gambling platforms (e.g., Suomi et al., 2024).

In summary, instant cash-out is a feature of concern within contemporary sports-betting apps, and the present study begins to shed light on the individuals for whom this feature is likely to be most appealing. Using a behavioral gambling task and a battery of self-report instruments in a general-population sample, we found that instant cash-out appeals most strongly to people who self-report less impulsivity and less hazardous gambling behavior. Our results therefore raise the concerning possibility that instant cash-out may serve as a gateway gambling product for less impulsive consumers.

## CRediT authorship contribution statement

**Ong George Ngieng:** Writing – review & editing, Writing – original draft, Formal analysis. **Lucy Albertella:** Writing – review & editing, Conceptualization. **Ty Hayes:** Writing – review & editing, Conceptualization. **Antonio Verdejo-Garcia:** Writing – review & editing, Conceptualization. **Lukasz Walasek:** Writing – review & editing, Conceptualization. **Elliot A. Ludvig:** Writing – review & editing, Conceptualization. **Daniel Bennett:** Writing – review & editing, Writing – original draft, Formal analysis, Data curation, Conceptualization.

## Declaration of competing interest

The authors declare that they have no known competing financial interests or personal relationships that could have appeared to influence the work reported in this paper.

## Data Availability

Data for the present study have been made freely available on the Open Science Framework
