# Supplementary material for: Greater impulsivity is associated with a reduced propensity to cash out of bets
Source: Addict Behav Rep. 2025 Nov 26;23:100645. doi: 10.1016/j.abrep.2025.100645 (PMC12702411; doi:10.1016/j.abrep.2025.100645)
Supplement: Supplementary Data 1 [file mmc1.pdf]

This document contains supplementary material associated with the following article published in *Addictive Behaviors Reports*:

Greater impulsivity is associated with a reduced propensity to cash out of bets

Ong George Ngieng[a], Lucy Albertella[a], Ty Hayes[b], Antonio Verdejo-Garcia[a], Lukasz Walasek[c], Elliot A. Ludvig[c] & Daniel Bennett[a,d]

[1] School of Psychological Sciences, Monash University, Australia

[2] Warwick Business School, University of Warwick, UK

[3] Department of Psychology, University of Warwick, UK

[4] Melbourne School of Psychological Sciences, The University of Melbourne, Australia

Supplementary Section S1: Further information on self-report questionnaires

Short UPPS-P. The Short UPPS-P (SUPPS-P; Cyders et al., 2014) is a 20-item short version of the UPPS-P Impulsive Behavior Scale (Lynam et al., 2006). In this scale, participants use a four-point Likert scale to rate their agreement with a series of statements (1 = disagree strongly; 4 = agree strongly). The SUPPS-P has five subscales: *Negative Urgency* (the tendency to behave impulsively while feeling negative emotion; example item: "When I am upset I often act without thinking"), *Positive Urgency* (the tendency to behave impulsively when feeling positive emotion; example item: "I tend to lose control when I am in a great mood"), *Lack of Premeditation* (the tendency to behave thoughtlessly or carelessly; example item (reverse-coded): "I like to stop and think things over before I do them"), *Lack of Perseverance* (the tendency to leave tasks unfinished; example item (reverse-coded): "I generally like to see things through to the end"), and *Sensation Seeking* (the tendency to seek out new and exciting experiences and sensations; example item: "I would like to learn to fly an airplane"). The SUPPS-P shows good internal consistency overall (α = .74-.88 across subscales), and has been linked with gambling cognitions and problem-gambling severity in previous studies (e.g., Tan & Tam, 2023). Numbering for individual items in the Results below are as per the full 59-item UPPS-P.

Dickman Impulsivity Inventory. The Dickman Impulsivity Inventory (DII; Dickman, 1990) is a 23-item self-report measure in which participants used a four-point Likert scale to rate their agreement with various statements (1 = disagree strongly; 4 = agree strongly). The DII has two subscales, respectively measuring *Dysfunctional Impulsivity* (i.e., impulsive behaviours that tend to produce negative or destructive consequences; example item: "I frequently buy things without thinking about whether or not I can really afford them") and *Functional Impulsivity* (i.e., impulsive behaviours that tend to produce positive or desirable consequences; example item: "I am good at taking advantage of unexpected opportunities, where you have to do something immediately or lose your chance"). Each of these subscales has good internal consistency (Dysfunctional Impulsivity: α = .87; Functional Impulsivity: α = .81; Pechorro et al., 2021) and both functional impulsivity and dysfunctional impulsivity have been found to be elevated in people who gamble frequently compared with the general population (Maccallum et al., 2007).

BIS/BAS. The Behavioral Inhibition System/Behavioral Approach System (BIS/BAS; Carver & White, 1994) is a 20-item self-report measure in which participants use a four-point Likert scale to rate their endorsement of a series of self-related statements (1 = very false for me;

4 = very true for me). The BIS/BAS comprises three BAS subscales: *Drive* (example item: “I go out of my way to get things I want”); *Fun Seeking* (example item: “I crave excitement and new sensations”); *Reward Responsiveness* (example item: “When I get something I want, I feel excited and energised”), and one *BIS* subscale (example item: “I feel worried when I think I have done poorly at something important”). There is mixed evidence concerning the external validity and factor structure of the measure (Demianczyk et al., 2014); nevertheless, we included the BIS/BAS scales in our battery because they are among the most widely used impulsivity-related self-report instruments to have been linked with gambling behaviour in previous research (e.g., Kim & Lee, 2011; Suhr & Tsanadis, 2007). In the present study, we omitted four “filler” items that were contained in the original 24-item BIS/BAS scale.

EDFLIX General Flexibility Subscale. The *General Flexibility* subscale of the Eating Disorder Flexibility Index (EDFLIX; Dahlgren et al., 2019) is a 17-item self-report measure of general cognitive and behavioural flexibility. In this subscale, participants use a six-point Likert scale (1 = strongly disagree, 6 = strongly agree) to rate their endorsement of a series of statements over the past 28 days, with scoring coded such that higher scores indicated higher levels of flexibility. Items included statements such as “I am open to new ways of doing things”, and “I find it difficult when something unexpected happens” [reverse-scored]). The full EDFLIX scale also includes two additional subscales specifically related to eating- and weight-related behaviours, neither of which was included in our questionnaire battery. The EDFLIX General Flexibility subscale has showed good internal consistency in previous research (α = .85; Liu et al., 2022). Although it has not previously been investigated in the context of gambling research, individual differences on this subscale have been shown to moderate the association between impulsivity and harm in related behavioural addictions such as Problematic Usage of the Internet (Liu et al., 2022).

Problem Gambling Severity Index. The Problem Gambling Severity Index (Ferris & Wynne, 2001) is a nine-item self-report measure of hazardous gambling behaviour that has shown good internal consistency in previous studies (α = .86; Miller et al., 2013), and is considered a gold-standard measure in the field (Dowling et al., 2018). Participants used a four-point Likert scale to rate the frequency of various gambling-related experiences over the past 12 months (0 = never; 3 = almost always). Items included statements such as “Have you bet more than you can really afford to lose?”, and “Has your gambling caused any financial problems for you or your household?”. Participants’ total PGSI scores were categorised according to standard cut-offs as *no*

risk of problem gambling (PGSI = 0), *low risk* (PGSI 1-2), *moderate risk* (PGSI 3-7), or "*problem gambler*" (PGSI 8+).

Full questionnaire materials are available in the project OSF repository.

Supplementary Section S1: Supplementary analyses of cash-out behaviour

We conducted a mixed-effects logistic regression to test which trial-level factors predicted higher cash-out rates across the sample as a whole. We found that participants' behaviour was sensitive to the value of the cash-out offer, with more generous cash-out offers more likely to be accepted (β = 0.61, SE = 0.06, p < .001; Figure S1A). There was no statistically significant main effect of either ambiguity (β = 0.06, SE = 0.04, p = .12) or Pr(win) (β = -0.03, SE = 0.16, p = .86; Figure S1B).

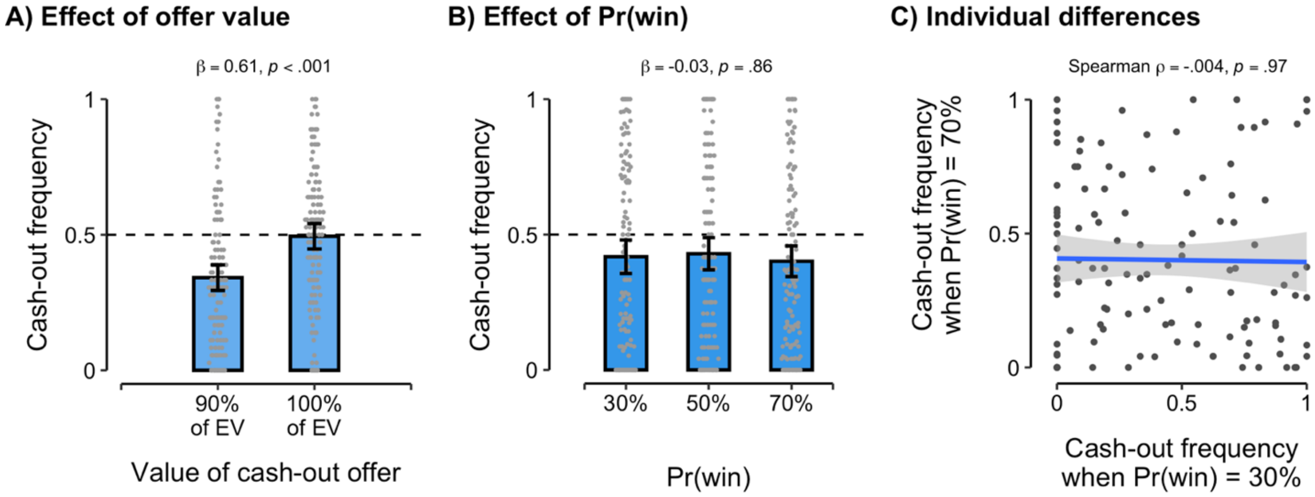

Figure S1. (A) The effect on cash-out frequency of cash-out offer value (defined as a percentage of bet expected value [EV]). Participants were significantly more likely to cash out of bets for which the cash-out offer value was equal to the EV of the bet. (B) There was no significant effect of bet win probability on cash-out frequency. (C) Participants' tendency to cash out of bets with declining odds (Pr(win) = 30%) was not associated with their tendency to cash out of bets with improving odds (Pr(win) = 70%). Subplots (A) and (B) depict the mean +/- the 95% confidence interval. The overlaid line on (C) depicts the linear association of best fit and its 95% confidence interval.

Replicating previous findings (Bennett et al., 2024), we found evidence of several significant two-way interactions: between Pr(win) and ambiguity ($\chi^2(1)$ = 29.53, p < .001) and between Pr(win) and cash-out offer value (β = 0.18, SE = 0.04, p < .001). For the interaction between

Pr(win) and ambiguity (Figure S2), post-hoc analyses run separately for low- and high-ambiguity trials indicated that these interactions may have been driven by a trend towards a negative association between Pr(win) and cash-out propensity when ambiguity was low (β = -0.31, SE = 0.16, p = .052) compared with a null effect of Pr(win) on cash-propensity when ambiguity was high (β = 0.23, SE = 0.16, p = .15).

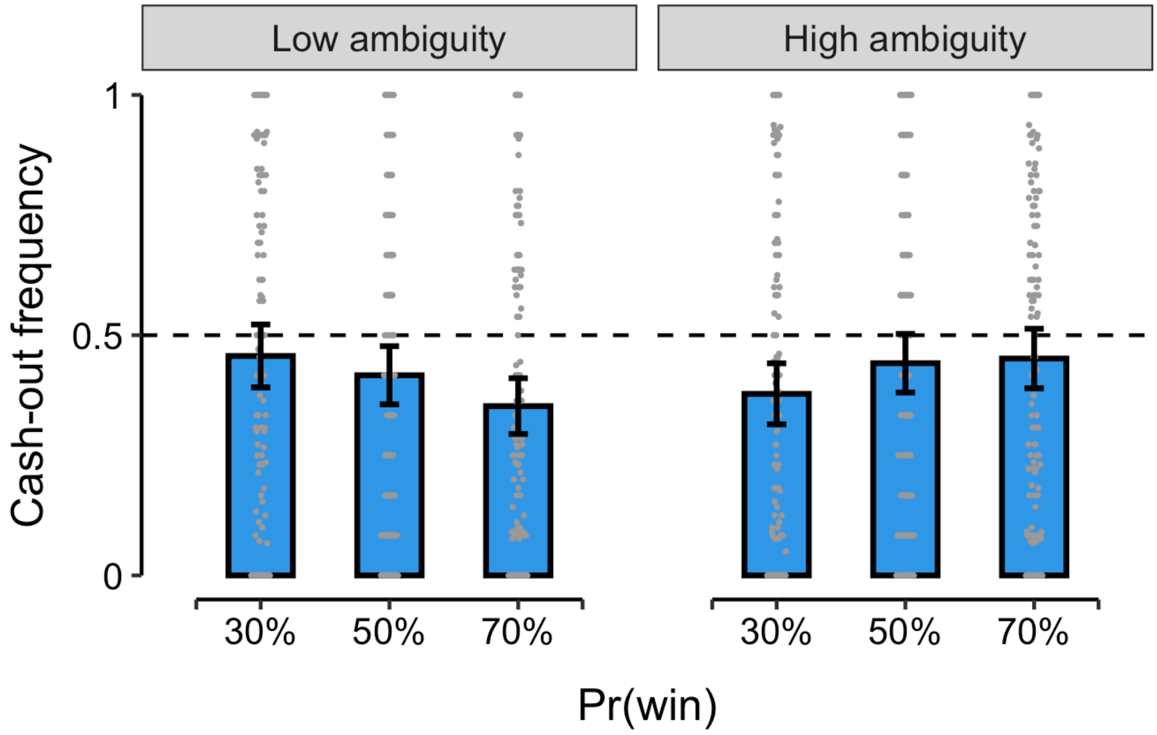

Figure S2. There was a significant interaction between Pr(win) (x-axis) and ambiguity (left/right facets). Points depict group-level means plus/minus the 95% confidence interval of the mean; grey points depict condition means for individual participants.

Post-hoc analyses also revealed that the interaction between Pr(win) and cash-out offer value was driven by a tendency for cash-out offer value to have a stronger effect on behaviour when Pr(win) was higher (Pr(win) = 70%: β = 0.87, $p < .001$; Pr(win) = 50%: β = 0.74, $p < .001$; Pr(win) = 30%: β = 0.39, $p < .001$). Figure S3 presents these results graphically.

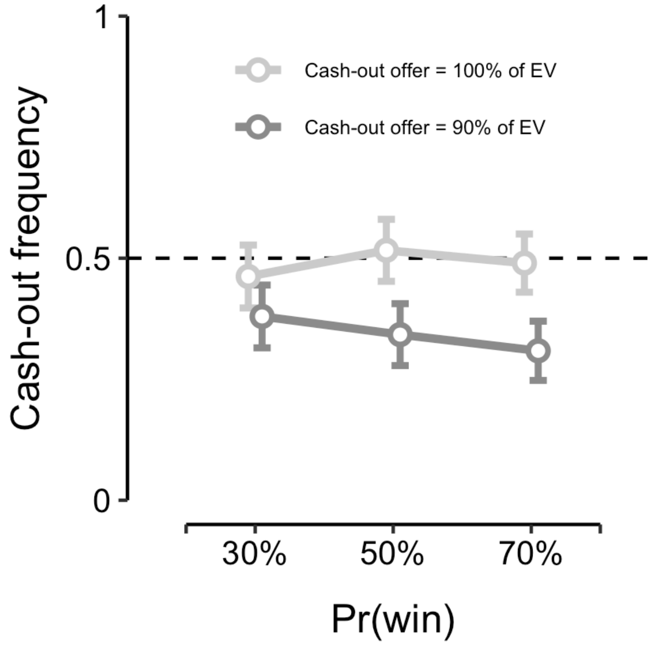

Figure S3. There was a significant interaction between Pr(win) (x-axis) and cash-out offer value (line colour). Points depict group-level means plus/minus the 95% confidence interval of the mean.

A complete regression model specification and full results are presented in Table S1 below.

Table S1a. Overview, mixed-effects logistic regressions of cash-out propensity

**Fixed effects**	**Participant-wise random effects**
- Intercept	- Random intercept
- Ambiguity (sum-coded)	- Random slopes for:
- Cash-out offer value (*z*-scored)	- Ambiguity (sum-coded)
- Pr(win) (*z*-scored)	- Cash-out offer value (*z*-scored)
- Ambiguity × cash-out offer value	- Pr(win) (*z*-scored)
- Ambiguity × Pr(win)	- Ambiguity × Pr(win)
- Offer value × Pr(win)	- Offer value × Pr(win)

Table S1b. Fixed-effect coefficient estimates for regression analysis

Omnibus effect	χ^2 (df)	*ß*	*p*	
Intercept	13.16 (1)	-0.78	< .001	***
Ambiguity	2.36 (1)	1.15	.12	
Cash-out offer value	120.31 (1)	0.82	< .001	***
Pr(win)	0.03 (1)	2.03	.86	
Ambiguity × cash-out offer value	0.0001 (1)	0.09	.99	
Ambiguity × Pr(win)	29.53 (1)	-3.49	< .001	***
Offer value × Pr(win)	17.98 (1)	0.19	< .001	***

Note: *** $p < .001$

Supplementary Section S2: Supplementary analyses of individual differences

Estimates of internal consistency for each of the self-report subscales included in this study are presented in Table S2 below.

Table S2. Cronbach's α for self-report subscales

Survey	Subscale	α	95% CI
BIS/BAS	BIS	.89	[.85, .91]
BIS/BAS	BAS Drive	.82	[.77, .87]
BIS/BAS	BAS Fun Seeking	.64	[.52, .73]
BIS/BAS	BAS Reward Responsiveness	.71	[.62, .78]
Dickman Impulsivity Inventory	Functional Impulsivity	.87	[.83, .90]
Dickman Impulsivity Inventory	Dysfunctional Impulsivity	.85	[.82, .89]
EDFLIX	General Flexibility	.91	[.88, .93]
SUPPS-P	Lack of Perseverance	.69	[.59, .77]
SUPPS-P	Lack of Premeditation	.85	[.80, .89]
SUPPS-P	Negative Urgency	.88	[.84, .91]
SUPPS-P	Positive Urgency	.88	[.84, .91]
SUPPS-P	Sensation Seeking	.79	[.71, .84]
PGSI	-	.92	[.89, .93]

Analyses of internal consistency (using split-half permutation testing with 1000 random permutations) revealed very strong participant-level internal consistency for overall cash-out frequency (estimated Spearman-Brown-corrected split-half reliability = .96, 95% CI = [.95, .97]). This high consistency indicates that the observed individual differences in cash-out behaviour reflected meaningful differences in preference between participants rather than behavioural noise or measurement error. Each of the three Pr(win) conditions also displayed strong internal consistency when split-half reliability was quantified separately by condition (30% Pr(win): split-

half reliability = .96, 95% CI = [.94, .97]; 50% Pr(win): reliability = .95, 95% CI = [.93, .96]; 70% Pr(win): reliability = .94, 95% CI = [.92, .96]).

In our previous analyses of individual differences in cash-out behaviour using a similar task, we found a negative association between cash-out frequency when Pr(win) = 30% and cash-out frequency when Pr(win) = 70% (Bennett et al., 2024). We did not replicate this negative association in the present study, but we did find evidence that cash-out frequency when Pr(win) = 30% was uncorrelated with cash-out frequency when Pr(win) = 70% (Spearman $\rho(121) = -.004$, $p = .97$). In other words, there was no association between a participant's likelihood of cashing out of a bet with deteriorating odds and their likelihood of cashing out of a bet with improving odds. This pattern suggests that cash-out tendencies in different Pr(win) conditions might be considered distinct behavioural phenotypes and also raises the possibility that behaviour in different Pr(win) conditions might display differential patterns of association with different impulsivity subscales.

Within-participant correlations of cash-out propensities at different levels of Pr(win) are presented in Table S3 below.

Table S3. Correlations in cash-out propensities at different levels of Pr(win)

Pr(win) condition	Pr(win) condition		
	30%	50%	70%
30%	-		
50%	.61**	-	
70%	-.004	.50**	-

Note: Spearman ρ correlations ($N = 123$). ** $p < .001$

We conducted a linear regression analysis to determine the extent to which individual differences in overall cash-out frequency could be predicted by participants' age, gender, and PGSI scores. Results of this analysis are presented in Table S4a for a four-way categorisation of PGSI scores according to standard cut-offs (coded as No Risk = 0, Low Risk = 1, Moderate Risk = 2, Problem Gambler = 3), and in Table S4b for a two-way categorisation of PGSI scores as either "No Risk" (PGSI = 0; coded as 0) or "At Risk" (PGSI > 0; coded as 1).

Table S4a. Coefficient estimates for PGSI regression analysis (four-way categorisation)

Omnibus effect	Contrast	*df*	*F (df)*	*ß*	*p*	
Intercept	-	(1, 118)	11.03	0.31	.001	**
Age	-	(1, 118)	4.15	0.01	.04	*
Gender	-	(2, 118)	0.27	-	.77	
	Non-binary (vs. man)	-	-	-0.04	.86	
	Woman (vs. man)	-	-	-0.03	.99	
PGSI	-	(1, 118)	5.87	-0.06	.02	*

Note: * $p < .05$; ** $p < .01$. Where both omnibus statistics and coefficients are estimated, reported *p*-values are for omnibus tests.

Table S4b. Coefficient estimates for PGSI regression analysis (two-way categorisation)

Omnibus effect	Contrast	*df*	*F (df)*	*ß*	*p*	
Intercept	-	(1, 118)	10.86	0.30	.001	**
Age	-	(1, 118)	4.39	0.01	.04	*
Gender	-	(2, 118)	0.29	-	.77	
	Non-binary (vs. man)	-	-	-0.04	.86	
	Woman (vs. man)	-	-	-0.03	.99	
PGSI	-	(1, 118)	5.40	-0.11	.02	*

Note: * $p < .05$; ** $p < .01$. Where both omnibus statistics and coefficients are estimated, reported *p*-values are for omnibus tests.

Cash-out frequency was significantly positively associated with age ($\rho(121) = .20$, $p = .03$; see Supplementary Figure S5F). All correlations between self-report subscales and behavioral cash-out frequency reported above remained statistically significant in partial correlations controlling for age (see Supplementary Table S5).

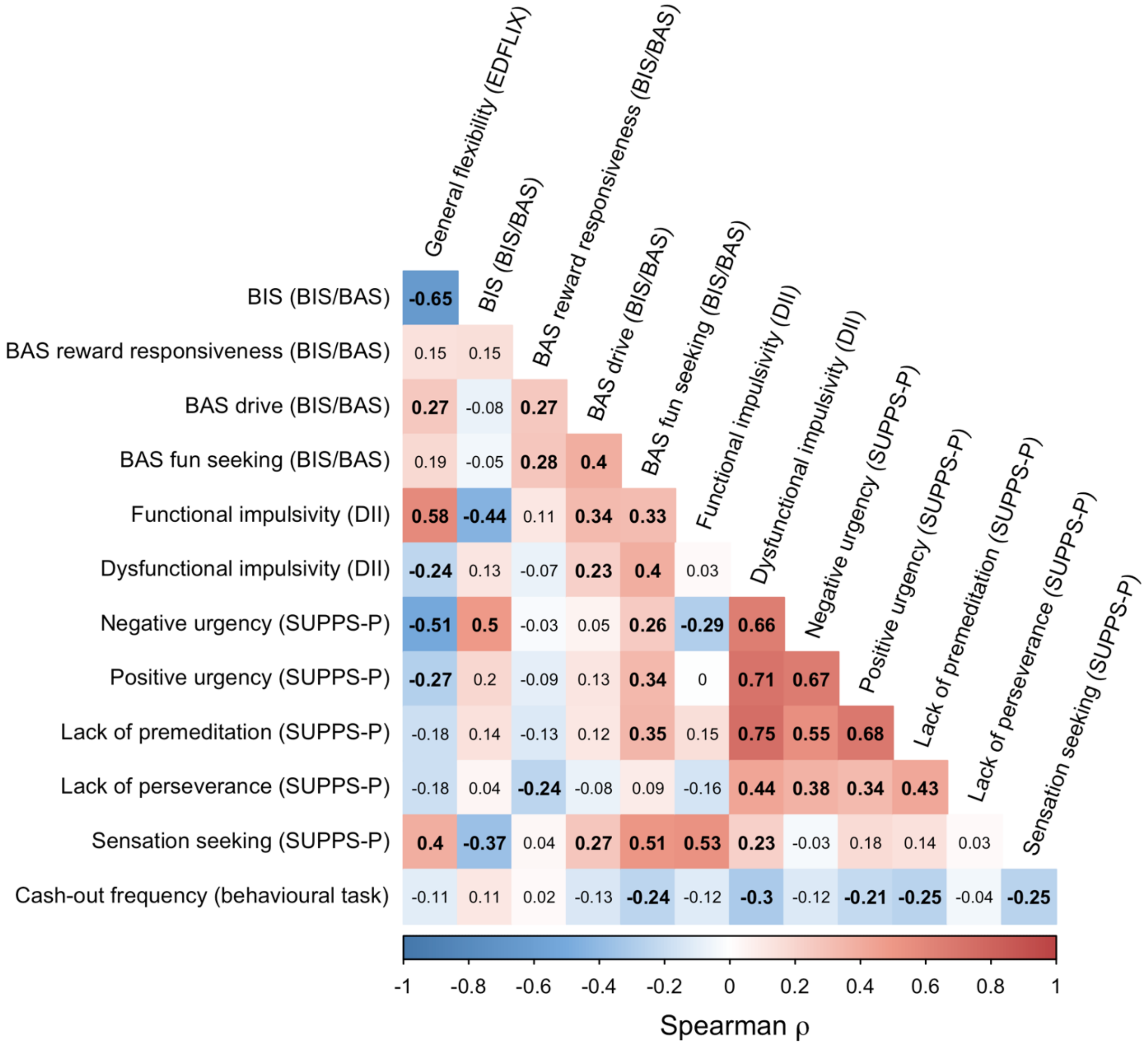

Figure S4. Spearman correlation matrix for associations between impulsivity subscales and overall cash-out frequency. Tile colour represents strength and direction of association; tiles with bold text represent correlations that were statistically significant after FDR correction for multiple comparisons.

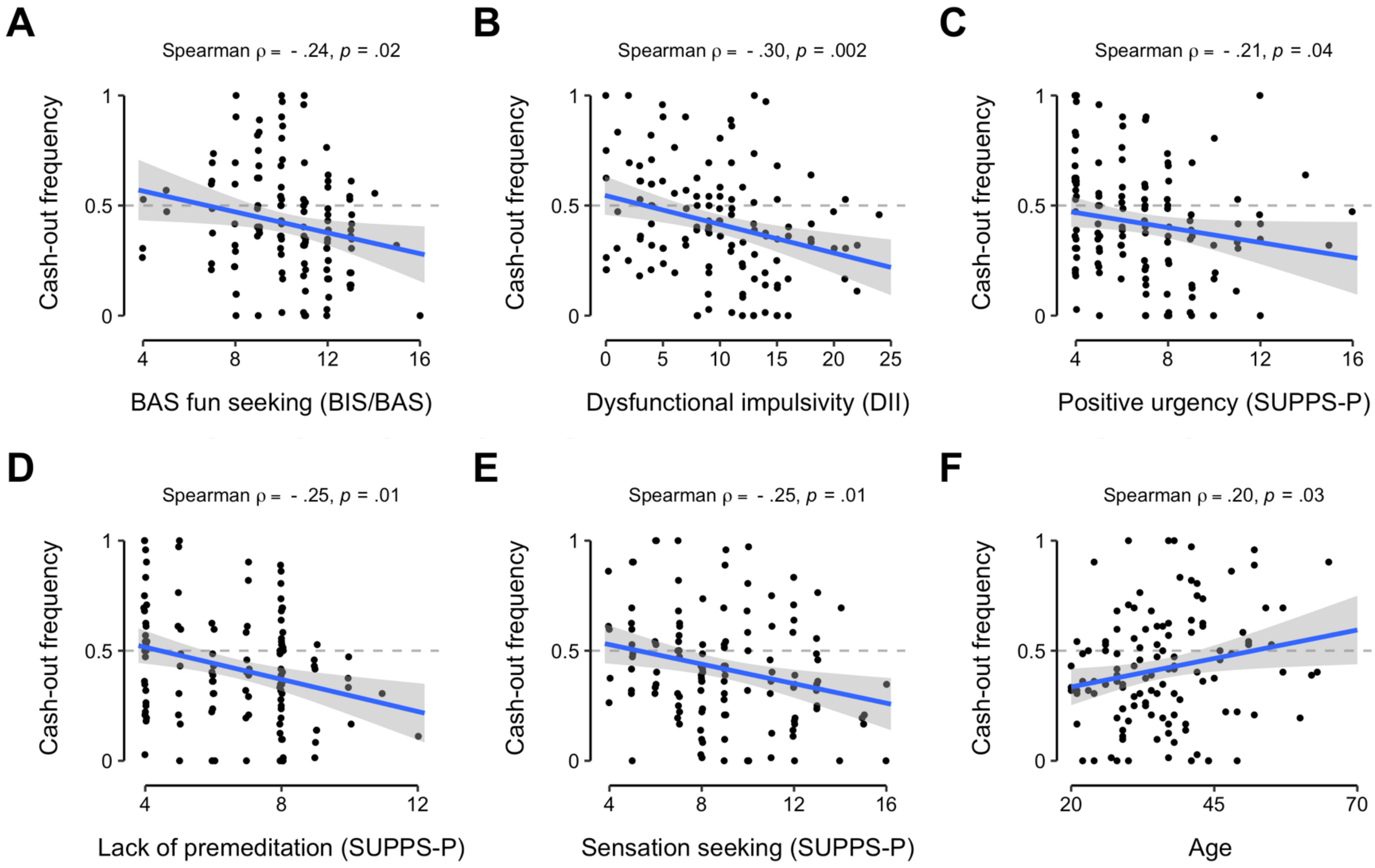

Figure S5. Scatterplots between individual differences in participants' survey self-report scores (x-axes) and their overall cash-out frequency in the behavioural task (y-axes; larger values indicate more frequent use of the cash-out feature). The overlaid lines represent the linear association of best fit and its 95% confidence. Values in subplots A – E have been horizontally jittered to prevent overplotting.

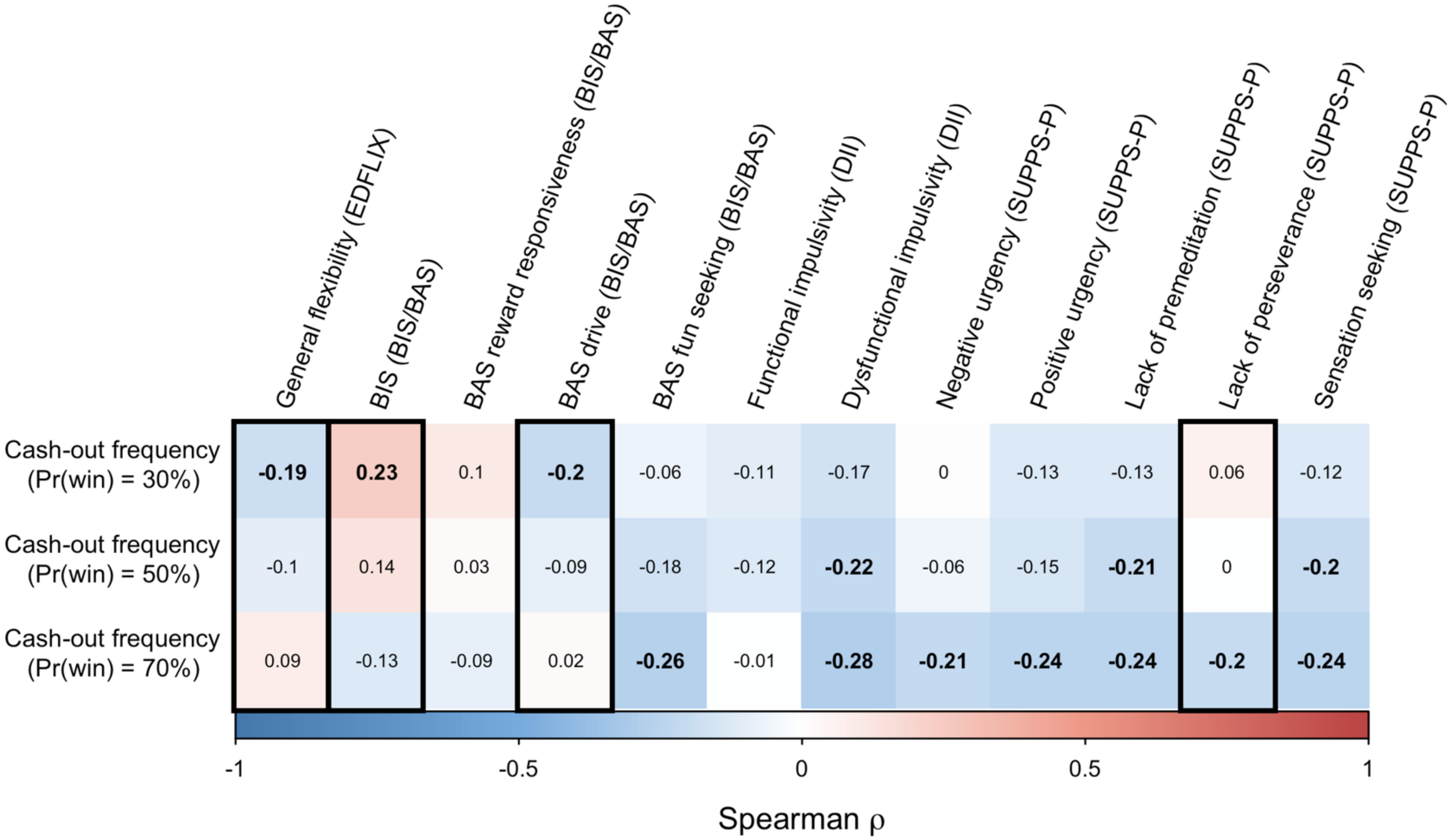

Figure S6. Spearman correlation matrix for associations between impulsivity subscales (columns) and cash-out frequency in different Pr(win) conditions (rows). Tile colour represents strength and direction of association; tiles with bold text represent correlations that were statistically significant after correction for multiple comparisons. Different patterns of association tended to occur across the different Pr(win) conditions; columns outlined in black are those for which the correlation between the impulsivity subscale and cash-out frequency was significantly moderated by Pr(win) according to a mixed-effects logistic regression analysis. EDFLIX: Eating Disorder Flexibility Index; BIS/BAS: Behavioural Inhibition System / Behavioural Activation System scale; DII: Dickman Impulsivity Inventory; SUPPS-P: Short Urgency-Premeditation-Perseverance-Sensation Seeking-Positive Urgency scale.

Table S5. Correlations between self-report subscales and behavioural cash-out frequency

Self-report subscale	Spearman correlation	
	Not controlling for age	Controlling for age
General flexibility (EDFLIX)	-.11	-.16
BIS (BIS/BAS)	.11	.16
BAS reward responsiveness	.02	.01
BAS drive	-.13	-.15
BAS fun seeking	-.24 *	-.22 *
Functional impulsivity (DII)	-.12	-.16
Dysfunctional impulsivity (DII)	-.30 **	-.28 **
Negative urgency (SUPPS-P)	-.12	-.09
Positive urgency (SUPPS-P)	-.21 *	-.18 *
Lack of premeditation (SUPPS-P)	-.25 *	-.23 *
Lack of perseverance (SUPPS-P)	-.04	-.04
Sensation seeking (SUPPS-P)	-.25 *	-.25 **

* $p < .05$; ** $p < .01$

Table S6. Correlations between self-report subscales and PGSI scores

Self-report subscale	Spearman correlation with PGSI
General flexibility (EDFLIX)	-.08
BIS (BIS/BAS)	.03
BAS reward responsiveness	.04
BAS drive	.20 *
BAS fun seeking	.29 **
Functional impulsivity (DII)	.05
Dysfunctional impulsivity (DII)	.27 **
Negative urgency (SUPPS-P)	.25 **
Positive urgency (SUPPS-P)	.23 *
Lack of premeditation (SUPPS-P)	.23 *
Lack of perseverance (SUPPS-P)	.12
Sensation seeking (SUPPS-P)	.14

* $p < .05$; ** $p < .01$

Supplementary Section S3: Supplementary factor-analytic results

The latent structure of participants' responses to self-report questionnaires was assessed using an exploratory factor analysis (EFA) of item-level responses. All item-level distributions displayed univariate normality across participants, and so an EFA was conducted on a Pearson correlation matrix. 16 items (out of 80 total, or 20%) were excluded due to a Kaiser-Meyer-Olkin (KMO) test score less than 0.6; the resulting Pearson correlation matrix was acceptable for EFA (KMO = 0.83 for total group of variables and 0.62 – 0.90 for each of the individual items). Parallel analysis and scree criteria were in agreement that four factors should be extracted. To preserve interpretability of the factor-loading matrix, items that would typically be reverse-scored for calculation of subscale totals were not reverse-scored within the exploratory factor analysis. Following inspection of item loadings, the four factors were identified as:

- Factor 1: a *Dysfunctional Impulsivity* factor primarily comprising items from the dysfunctional impulsivity subscale of the DII (e.g., DII-18: "I often get into trouble because I don't think before I act"; factor loading = .84), plus items from the Positive Urgency, Negative Urgency, and Lack of Premeditation subscales of the SUPPS-P (e.g., SUPPS-P-20: "I tend to lose control when I am in a great mood"; factor loading = .82). Coefficient alpha for this factor was .95 (95% CI = .93-.96). Factor 1 explained 17% of the total variance in the data and 37% of the common variance.

- Factor 2: an *Inhibition and Inflexibility* factor primarily comprising reverse-coded items from the general flexibility subscale of the EDFLIX (e.g., EDFLIX-17: "I find it difficult when something unexpected happens"; factor loading = .73) as well as items from the Behavioral Inhibition Scale of the BIS/BAS (e.g., BIS/BAS-19: "I feel worried when I think I have done poorly at something important"; factor loading = .76). Coefficient alpha for this factor was .92 (95% CI = .90-.94). Factor 2 explained 13% of the total variance in the data and 28% of the common variance.

- Factor 3: a *Reward Responsiveness* factor comprising items from the Functional Impulsivity subscale of the DII (e.g., DII-02: "I am good at taking advantage of unexpected opportunities, where you have to do something immediately or lose your

chance"; factor loading = .60) as well as several other items related to reward-seeking behaviour (e.g., BISBAS-03: "When I'm doing well at something I love to keep at it"; factor loading = .58 and SUPPS-P-46: "I would enjoy the feeling of skiing very fast down a high mountain slope"; factor loading = .57). Coefficient alpha for this factor was .87 (95% CI = .84-.90). Factor 3 explained 9% of the total variance in the data and 20% of the common variance.

- Factor 4: an *Adaptability* factor composed of items from the general flexibility subscale of the EDFLIX (e.g., EDFLIX-23: "If I have to, it's easy for me to change my plans"; factor loading = .70). Coefficient alpha for this factor was .88 (95% CI = .84-.91). Factor 4 explained 7% of the total variance in the data and 15% of the common variance.

These four factors cumulatively explained 46% of the total variance in the data. A factor correlation matrix is presented in Supplementary Table S7, and factor loadings and communalities for each of the 64 self-report items included in the factor analysis are presented in Supplementary Table S8. Participants' PGSI scores were significantly positively associated with their scores on the Dysfunctional Impulsivity factor (Spearman $\rho(121) = .29$, $p = .001$) and the Reward Responsiveness factor (Spearman $\rho(121) = .19$, $p = .04$), but not with the Inhibition and Inflexibility or Adaptability factor scores (both $p > .10$).

Table S7. Factor correlation matrix

	Factor 1	Factor 2	Factor 3	Factor 4
Factor 1: *Dysfunctional impulsivity*	1	.33	.14	-.17
Factor 2: *Inhibition and inflexibility*	.33	1	-.35	-.20
Factor 3: *Reward responsivity*	.14	-.35	1	.47
Factor 4: *Adaptability*	-.17	-.20	.47	1

Table S8. Communalities and factor loadings for individual self-report items including in factor analysis

Scale item	Original subscale	Item text	Factor 1	Factor 2	Factor 3	Factor 4	Communality
BIS/BAS-02	BIS	Even if something bad is about to happen to me, I rarely experience fear or nervousness	-	**-.54**	-	-	.44
BIS/BAS-03	BAS drive	I go out of my way to get things I want	-	**-**	**.58**	-	.43
BIS/BAS-08	BIS	Criticism or scolding hurts me quite a bit	-	**.71**	-	-	.51
BIS/BAS-10	BAS fun seeking	I will often do things for no other reason that that they might be fun	-	-	-	-	.26
BIS/BAS-14	BAS reward responsiveness	When I see an opportunity for something I like I get excited right away	-	-	**.42**	-	.34
BIS/BAS-15	BAS fun seeking	I often act on the spur of the moment	**.64**	-	-	-	.45
BIS/BAS-16	BIS	If I think something unpleasant is going to happen I usually get pretty 'worked up'	-	**.62**	-	-	.37
BIS/BAS-19	BIS	I feel worried when I think I have done poorly at something important	-	**.76**	-	-	.52
BIS/BAS-20	BAS fun seeking	I crave excitement and new sensations	-	-	**.44**	-	.32
BIS/BAS-21	BAS drive	When I go after something I use a 'no holds barred' approach	-	-	-	-	.22
BIS/BAS-22	BIS	I have very few fears compared to my friends	-	**-.43**	-	-	.46
BIS/BAS-24	BIS	I worry about making mistakes	-	**.64**	-	-	.47
DII-01	Functional impulsivity	I don't like to make decisions quickly, even simple decisions such as choosing what to wear or what to have for dinner	-	-	-	-	.40
DII-02	Functional impulsivity	I am good at taking advantage of unexpected opportunities, where you have to do something immediately or lose your chance	-	-	**.60**	-	.49
DII-03	Functional impulsivity	Most of the time, I can put my thoughts into words very rapidly	-	-	**.57**	-	.32
DII-04	Functional impulsivity	I am uncomfortable when I have to make up my mind rapidly	-	**.44**	**-.50**	-	.53
DII-05	Functional impulsivity	I like to take part in really fast-paced conversations, where you don't have much time to think before you speak	-	-	**.49**	-	.33

Scale item	Original subscale	Item text	Factor 1	Factor 2	Factor 3	Factor 4	Communality
DII-07	Functional impulsivity	I would enjoy working at a job that required me to make a lot of split-second decisions	-	-	**.55**	-	.57
DII-08	Functional impulsivity	I like sports and games in which you have to choose your next move very quickly	-	-	**.70**	-	.43
DII-09	Functional impulsivity	I have often missed out on opportunities because I couldn't make up my mind fast enough	-	-	-	-	.33
DII-10	Functional impulsivity	People have admired me because I can think quickly	-	-	**.66**	-	.33
DII-11	Functional impulsivity	I try to avoid activities where you have to act without too much time to think	-	**.42**	**-.58**	-	.61
DII-12	Dysfunctional impulsivity	I will often say whatever comes into my head without thinking first	**.70**	-	-	-	.57
DII-14	Dysfunctional impulsivity	I frequently make appointments without thinking about whether or not I will be able to keep them	**.55**	-	-	-	.42
DII-15	Dysfunctional impulsivity	I frequently buy things without thinking about whether or not I can really afford them	**.58**	-	-	-	.38
DII-16	Dysfunctional impulsivity	I often make up my mind without taking the time to consider the situation from all angles	**.68**	-	-	-	.45
DII-17	Dysfunctional impulsivity	Often, I don't spend enough time thinking over a situation before I act	**.68**	-	-	-	.43
DII-18	Dysfunctional impulsivity	I often get into trouble because I don't think before I act	**.84**	-	-	-	.66
DII-21	Dysfunctional impulsivity	Before making any important decision, I carefully weigh the pros and cons	**-.47**	-	-	-	.23
DII-22	Dysfunctional impulsivity	I am good at careful reasoning	**-.50**	-	-	-	.32
DII-23	Dysfunctional impulsivity	I often say and do things without considering the consequences	**.74**	-	-	-	.65
EDFLIX-04	General flexibility	I think I handle changes well	-	**-.52**	-	**.56**	.73
EDFLIX-05	General flexibility	I find it difficult to get used to new situations	-	**.44**	-	-	.57
EDFLIX-06	General flexibility	Sudden changes make me distressed	-	**.60**	-	-	.70
EDFLIX-11	General flexibility	I find it easy to do several things at once	-	-	**.41**	-	.22
EDFLIX-13	General flexibility	When I am stuck on a task, I am unable to come up with new solutions	-	-	-	-	.26
EDFLIX-14	General flexibility	It bothers me when things don't go exactly as planned	-	**.63**	-	-	.45

Scale item	Original subscale	Item text	Factor 1	Factor 2	Factor 3	Factor 4	Communality
EDFLIX-16	General flexibility	It's easy for me to adapt to changes in my environment (e.g., a new workplace / school / home / new friends/colleagues, etc.)	-	-	-	**.69**	.71
EDFLIX-17	General flexibility	I get anxious or distressed if others interfere with my plans	-	**.73**	-	-	.57
EDFLIX-21	General flexibility	I am open to new ways of doing things	-	-	-	**.47**	.39
EDFLIX-23	General flexibility	If I have to, it's easy for me to change my plans	-	-	-	**.70**	.58
EDFLIX-25	General flexibility	I find it difficult when something unexpected happens	-	**.69**	-	-	.68
EDFLIX-27	General flexibility	I find it difficult to consider a situation from several perspectives	-	-	-	-	.32
EDFLIX-30	General flexibility	I am usually quite flexible	-	-	-	**.68**	.60
EDFLIX-31	General flexibility	When things don't go to plan, I am able to consider alternative solutions	-	-	-	**.55**	.43
EDFLIX-35	General flexibility	I get angry or upset when people don't do things my way	-	**.65**	-	-	.52
SUPPS-P-04	Lack of perseverance	I generally like to see things through to the end	**-.41**	-	-	-	.26
SUPPS-P-06	Lack of premeditation	My thinking is usually careful and purposeful	**-.67**	-	-	-	.53
SUPPS-P-10	Positive urgency	When I am in a great mood, I tend to get into situations that could cause me problems	**.82**	-	-	-	.59
SUPPS-P-16	Lack of premeditation	I like to stop and think things over before I do them	**-.69**	-	-	-	.49
SUPPS-P-17	Negative urgency	When I feel bad, I will often do things I later regret in order to make myself feel better now	**.58**	-	-	-	.58
SUPPS-P-20	Positive urgency	I tend to lose control when I am in a great mood	**.82**	-	-	-	.58
SUPPS-P-22	Negative urgency	Sometimes when I feel bad, I can't seem to stop what I am doing even though it is making me feel worse	**.51**	-	-	-	.57
SUPPS-P-23	Sensation seeking	I quite enjoy taking risks	-	-	**.59**	-	.63
SUPPS-P-27	Lack of perseverance	I finish what I start	-	-	-	-	.32
SUPPS-P-28	Lack of premeditation	I tend to value and follow a rational, 'sensible' approach to things	**-.57**	-	-	-	.38
SUPPS-P-29	Negative urgency	When I am upset I often act without thinking	**.68**	-	-	-	.64

Scale item	Subscale	Item text	Factor 1	Factor 2	Factor 3	Factor 4	Communality
SUPPS-P-31	Sensation seeking	I welcome new and exciting experiences and sensations, even if they are a little frightening and unconventional	-	-	-	-	.38
SUPPS-P-34	Negative urgency	When I feel rejected, I often say things that I later regret	**.51**	-	-	-	.54
SUPPS-P-35	Positive urgency	Others are shocked or worried about the things I do when I am feeling very excited	**.78**	-	-	-	.52
SUPPS-P-36	Sensation seeking	I would like to learn to fly an airplane	-	-	-	-	.20
SUPPS-P-46	Sensation seeking	I would enjoy the feeling of skiing very fast down a high mountain slope	-	-	**.57**	-	.31
SUPPS-P-48	Lack of premeditation	I usually think carefully before doing anything	**-.70**	-	-	-	.32
SUPPS-P-52	Positive urgency	I tend to act without thinking when I am really excited	**.72**	-	-	-	.65

Absolute factor loadings > .40 are shown. Item numbers are per the original publication of each scale

Finally, Table S9a presents full details of the fixed- and random-effects structure of the mixed-effects logistic regression of cash-out including factor scores. Full regression results for this model are presented in Table S9b below.

Table S9a. Overview, mixed-effects logistic regression of cash-out propensity with factor scores

**Fixed effects**	**Participant-wise random effects**
- Intercept<br>- Pr(win) (*z*-scored)<br>- Dysfunctional Impulsivity factor score (z-scored)<br>- Inhibition and Inflexibility factor score (z-scored)<br>- Reward Responsiveness factor score (z-scored)<br>- Adaptability factor score (z-scored)<br>- Pr(win) × Dysfunctional Impulsivity<br>- Pr(win) × Inhibition and Inflexibility<br>- Pr(win) × Reward Responsiveness<br>- Pr(win) × Adaptability	- Random intercept<br>- Random slope for:<br>- Pr(win) (*z*-scored)

Table S9b. Fixed-effect coefficient estimates for logistic regression with factor scores

Coefficent	*ß* (SE)	*p*	
Intercept	-0.64 (0.18)	< .001	***
Pr(win)	0.03 (0.13)	.84	
Dysfunctional Impulsivity	-0.52 (0.21)	.02	*
Inhibition and Inflexibility	0.33 (0.22)	.13	
Reward Responsiveness	-0.34 (0.25)	.17	
Adaptability	0.08 (0.16)	.73	
Pr(win) × Dysfunctional Impulsivity	0.09 (0.16)	.58	
Pr(win) × Inhibition and Inflexibility	-0.45 (0.16)	.006	**
Pr(win) × Reward Responsiveness	-0.21 (0.18)	.23	
Pr(win) × Adaptability	0.22 (0.17)	.18	

Note: *** *p* < .001; ** *p* < .01; * *p* < .05

References for Supplementary Material

Bennett, D., Albertella, L., Forbes, L., Hayes, T., Verdejo-Garcia, A., Walasek, L., & Ludvig, E. A. (2024). People Place Larger Bets When Risky Choices Provide a Postbet Option to Cash Out. *Psychological Science*, *35*(11), 1231–1245. https://doi.org/10.1177/09567976241266516

Carver, C. S., & White, T. L. (1994). Behavioral inhibition, behavioral activation, and affective responses to impending reward and punishment: The BIS/BAS scales. *Journal of Personality and Social Psychology*, *67*(2), 319–333.

Cyders, M. A., Littlefield, A. K., Coffey, S., & Karyadi, K. A. (2014). Examination of a short English version of the UPPS-P Impulsive Behavior Scale. *Addictive Behaviors*, *39*(9), 1372–1376. https://doi.org/10.1016/j.addbeh.2014.02.013

Dahlgren, C. L., Hage, T. W., Wonderlich, J. A., & Stedal, K. (2019). General and Eating Disorder Specific Flexibility: Development and Validation of the Eating Disorder Flexibility Index (EDFLIX) Questionnaire. *Frontiers in Psychology*, *10*, 663. https://doi.org/10.3389/fpsyg.2019.00663

Demianczyk, A. C., Jenkins, A. L., Henson, J. M., & Conner, B. T. (2014). Psychometric Evaluation and Revision of Carver and White's BIS/BAS Scales in a Diverse Sample of Young Adults. *Journal of Personality Assessment*, *96*(5), 485–494. https://doi.org/10.1080/00223891.2013.870570

Dickman, S. J. (1990). Functional and dysfunctional impulsivity: Personality and cognitive correlates. *Journal of Personality and Social Psychology*, *58*(1), 95–102. https://doi.org/10.1037/0022-3514.58.1.95

Dowling, N. A., Merkouris, S. S., Manning, V., Volberg, R., Lee, S. J., Rodda, S. N., & Lubman, D. I. (2018). Screening for problem gambling within mental health services: A comparison of the classification accuracy of brief instruments. *Addiction*, *113*(6), 1088–1104. https://doi.org/10.1111/add.14150

Ferris, J., & Wynne, H. (2001). *The Canadian Problem Gambling Index: Final Report*. Canadian Consortium for Gambling Research.

Kim, D.-Y., & Lee, J.-H. (2011). Effects of the BAS and BIS on decision-making in a gambling task. *Personality and Individual Differences*, *50*(7), 1131–1135. https://doi.org/10.1016/j.paid.2011.01.041

Liu, C., Rotaru, K., Chamberlain, S. R., Ren, L., Fontenelle, L. F., Lee, R. S. C., Suo, C., Raj, K., Yücel, M., & Albertella, L. (2022). The Moderating Role of Psychological Flexibility on the Association between Distress-Driven Impulsivity and Problematic Internet Use. *International Journal of Environmental Research and Public Health*, *19*(15), 9592. https://doi.org/10.3390/ijerph19159592

Lynam, D. R., Smith, G. T., Whiteside, S. P., & Cyders, M. A. (2006). *The UPPS-P: Assessing five personality pathways to impulsive behavior*. Purdue University.

Maccallum, F., Blaszczynski, A., Ladouceur, R., & Nower, L. (2007). Functional and dysfunctional impulsivity in pathological gambling. *Personality and Individual Differences*, *43*(7), 1829–1838. https://doi.org/10.1016/j.paid.2007.06.002

Miller, N. V., Currie, S. R., Hodgins, D. C., & Casey, D. (2013). Validation of the Problem Gambling Severity Index using Confirmatory Factor Analysis and Rasch Modelling. *International Journal of Methods in Psychiatric Research*, *22*(3), 245–255. https://doi.org/10.1002/mpr.1392

Pechorro, P., Revilla, R., Resende, M., Abrunhosa Gonçalves, R., Nunes, C., & Cyders, M. A. (2021). The Dickman Impulsivity Inventory: Validation and measurement invariance among Portuguese young adults. *PLOS ONE*, *16*(12), e0260621. https://doi.org/10.1371/journal.pone.0260621

Suhr, J. A., & Tsanadis, J. (2007). Affect and personality correlates of the Iowa Gambling Task. *Personality and Individual Differences*, *43*(1), 27–36. https://doi.org/10.1016/j.paid.2006.11.004

Tan, G. S. L., & Tam, C. L. (2023). Impulsivity, Gambling-Related Cognitions, Cognitive Reappraisal and Gambling Behaviour in a Malaysian Sample. *Journal of Gambling Studies*, *40*(2), 475–492. https://doi.org/10.1007/s10899-023-10246-7
